# Survival risk prediction model for patients with pT_1–3_ N_0_M_0_ esophageal squamous cell carcinoma after R0 esophagectomy with two-field lymphadenectomy for therapeutic purposes

**DOI:** 10.1186/s13019-021-01503-0

**Published:** 2021-05-01

**Authors:** Zhan Qi, Yuanping Hu, Rong Qiu, Juan Li, Yuekao Li, Ming He, Yuxiang Wang

**Affiliations:** 1 Department of thoracic surgery, Fourth Hospital of Hebei Medical University, Shijiazhuang, 050011 China; 2 Department of Radiation Oncology, Fourth Hospital of Hebei Medical University, No.12, Jiankang road, Shijiazhuang, 050011 China; 3 Hebei Clinical Research Center for Radiation Oncology, Shijiazhuang, China; 4 Department of CT/MRI, Fourth Hospital of Hebei Medical University, Shijiazhuang, 050011 China

**Keywords:** Esophageal squamous cell carcinoma, Extended esophagectomy with two-field lymphadenectomy, Recursive partitioning analysis, Survival

## Abstract

**Background:**

The overall survival (OS) remains unsatisfactory in patients with esophageal squamous cell carcinoma (ESCC) after extended esophagectomy with two-field lymphadenectomy. Therefore, this retrospective study aimed to identify the risk factors that contribute to the low survival of patients with pT_1–3_N_0_M_0_ ESCC.

**Methods:**

Patients with pT_1–3_N_0_M_0_ ESCC who only underwent R0 esophagectomy with two-field lymphadenectomy in our department from January 2008 to December 2012 were retrospectively enrolled in this study and medical records were reviewed. Postoperative OS, disease-free survival (DFS), recurrence-free survival (RFS), and locoregional recurrence-free survival (LRFS) were analyzed sequentially.

**Results:**

This study recruited a total of 488 patients, whose follow-up visits were completed at the end of December 2019. The five-year OS, DFS, RFS and LRFS rates were 62.1, 53.1, 58.3 and 65.6%, respectively. Multivariate Cox analysis identified patient age, site of the lesion, small mediastinal lymph nodes in CT imaging (SLNs in CT), dissected lymph nodes (LNs), and stage of esophageal malignancy as independent risk factors for OS of the patients. Of these factors, the site of the lesion, SLNs in CT and stage of the cancer were determined to be independent factors for DFS, RFS and LRFS. Based on all five factors, the recursive partitioning analysis (RPA) score system was developed to stratify the patients into low-, medium- and high-risk groups, which were found to possess significantly different rates of OS, DFS, RFS and LRFS (*p* < 0.001).

**Conclusions:**

Several factors were associated with the survival of patients with pT_1–3_ N_0_M_0_ ESCC who underwent extended esophagectomy with two-field lymphadenectomy. These factors contributed to the RPA scoring system, which could stratify the risk of postoperative survival and may expedite the initiation of postoperative adjuvant therapy.

## Background

Esophageal cancer (EC) ranked seventh in terms of incidence and sixth in overall mortality in 2018 [1]. Esophageal squamous cell carcinoma (ESCC) is the predominant pathological type of EC worldwide [2]. The Ivor-Lewis subtotal esophagectomy with either two-field or three-field lymphadenectomy remains the current procedure of choice for resectable ESCC, but the postoperative prognosis remains unsatisfactory at only 50% for five-year overall survival (OS) in patients with pathologic negative lymph node (pN_0_) ESCC [3–5]. Postoperative recurrence, especially locoregional recurrence (LRR), has been shown to be relatively high in pN_0_ ESCC patients [6–8].

Moreover, the effectiveness of postoperative adjuvant chemo- and radiation therapy has not been established for the management of pN_0_ ESCC patients [8–10]. Previous studies have shown that several factors, such as age, tumor location, the number of dissected lymph nodes (LNs), pathological stage, and others were associated with the survival of ESCC patients after surgery [3–14]. A recursive partitioning analysis (RPA)-based system has been used to evaluate and predict the prognosis of EC patients after resection [15–17].

This retrospective study aimed to identify the factors associated with a survival benefit, and to further stratify the survival risk based on the RPA scores for patients with stage pN_0_ ESCC following two-field esophagectomy. These findings will help to determine the need for postoperative adjuvant therapy in these patients to improve OS.

## Methods

### Patients

All patients in this study completed a full course of follow-up visits in 2016 and were recruited for this second round of additional study. The inclusion criteria were described as follows: (1) patients underwent radical (R0) esophagectomy with two-field lymphadenectomy in our hospital between January 2008 and December 2012; (2) patients had a pathological diagnosis of ESCC; (3) patients were staged as pT_1–3_N_0_M_0_; (4) patients were not managed with either neoadjuvant or adjuvant therapy; (5) patients were not found to have a history of other malignancies; (6) the postoperative survival time was at least 3 months to minimize the impact of surgical complications on survival; and (7) patients had participated and were followed-up in our previous study [6]. The exclusion criteria were: (1) patients had non-ESCC at their pathological diagnosis; (2) patients underwent either R_1_ or R_2_ resection or three-field lymphadenectomy; (3) patients were pathologically staged as either pT_4_, pN_+_ or M_1_; (4) patients were managed with either pre- and/or post-operative adjuvant therapy; and (5) post-operative survival time was less than 3 months.

The study design and related ethical issues were approved by the Medical Ethics Committee of our hospital, and written informed consent was obtained from all patients enrolled in this study. Furthermore, the medical records of all patients were reviewed and information pertinent to the study was extracted from the records, including patient demographics, perioperative work-ups, detailed intraoperative information, and information related to postoperative management as well as long-term follow-ups.

### Surgery

The left thoracic approach (Sweet procedure) was routinely chosen to gain access to the primary tumors located in the middle/lower thoracic segment of the esophagus. The right thoracic approach (Ivor Lewis procedure) was used to access the primary tumors located in the upper thoracic segment of the esophagus. Radical surgical resection consisted of transthoracic subtotal esophagectomy with abdominal and mediastinal lymphadenectomy. A gastric tube placed through the posterior mediastinal route was used as a substitute for the resected esophagus to restore the continuity of the alimentary tract. Two-field LN dissection included total mediastinal, perigastric, and celiac lymphadenectomy.

### Follow-up

The first postoperative follow-up visits were scheduled for 1 month after the surgery. Thereafter, patients were sequentially followed every 3 months for the first 2 years, every 6 months for the next 3 years, and then every 12 months. The deadline for all follow-ups was December 1st, 2019. During the follow-up visits, patients were re-examined with chest computed tomography (CT) scans, and abdominal and cervical ultrasounds or CTs. When necessary, endoscopy, radionuclide bone imaging, or positron emission tomography (PET)/CT scans were also offered to the patients.

### Statistical analysis

In this study, overall survival (OS) was defined as the period from the date of surgery to the date of death or last follow-up. Disease-free survival (DFS) was defined as the time from the date of surgery to the date of the first evidence of recurrence or death of any cause. Recurrence-free survival (RFS) was defined as the period from the date of surgery to the date of the first evidence of tumor recurrence. Locoregional recurrence-free survival (LRFS) was defined as the period from the date of surgery to the date of the first evidence of locoregional recurrence (LRR) of the malignancy. LRR was defined as neoplastic recurrence at the original cancer site or stoma area, or appearance of metastatic lymph nodes in the supraclavicular, mediastinum, or epigastrium regions. Relapses at other sites were defined as distant metastases (DM).

The survival rate was calculated using the Kaplan-Meier method, and comparisons between groups were performed with the log-rank test. A two-tailed *p* value < 0.05 was considered statistically significant. Multivariate Cox regression analyses were performed to identify prognostic factors for survival. All statistical analyses were conducted using SPSS 22.0 software (IBM Corp, Armonk, NY, USA).

## Results

### Patient demographics

A total of 488 patients with pT_1–3_N_0_M_0_ thoracic ESCC were enrolled in this study. The median age was 62 years (range: 34–86 years) and the ratio of males to females was 1.64:1. Preoperative imaging determined that the median tumor size was 4 cm (range: 1–10 cm) and mediastinal small LNs (transverse section diameter < 1 cm) were visualized in 115 patients (defined as “SLNs in CT” below). Intraoperatively, two-field lymphadenectomy was used to dissect out local LNs (defined as “dissected LNs” below) and the median number removed was 10 (range: 1–27; Table 1).

**Table 1:** Clinical characteristics of pT_1 − 3_N_0_M_0_ ESCC patients

Characteristics	Number (%)	Characteristicss	Number (%)
Gender		Anastomotic sites	
Male	303 (62.1%)	Neck	49 (10.1%)
Female	185 (37.9%)	Above aortic arch	394 (80.7%)
History of smoking	209 (42.8%)	Below aortic arch	45 (9.2%)
Alcohol consumption	158 (32.3%)	Dissected LNs	
SLNs in CT	115 (23.6%)	< 12	337 (69.1%)
Site of lesion		≥ 12	151 (30.9%)
Upper	61 (12.5%)	Differentiation of ESCC	
Middle	344 (70.5%)	Well/moderate	450 (92.2%)
Lower	83 (17.0%)	Poor	38 (7.8%)
Surgical approach		pT	
Left thoracic	439 (90%)	pT_1_N_0_M_0_	102 (20.9%)
Right thoracic	49 (10%)	pT_2_N_0_M_0_	126 (25.8%)
		pT_3_N_0_M_0_	260 (53.3%)

4 cm (range from 1 to 10 cm)

*Abbreviations and definitions*: *ESCC* esophageal squamous cell carcinoma, *dissected LNs* the number of dissected lymph nodes at the time of surgery, *SLNs in CT* small lymph nodes in mediastinum (diameter < 1 cm) in CT image prior to surgery

### Outcomes

Among 488 patients, 226 had a recurrence of their cancer for an overall recurrence rate of 46.3%. The recurrence developed locally in 182 patients, and the LRR rate was 37.3%. Moreover, 213 (43.6%) patients died during the follow-up period, of which 173 (35.5%) were the result of tumor progression and 40 (8.2%) were due to non-cancer-related causes. Finally, 36 patients were lost to follow-up before the deadline of December 1st, 2019, which gave a follow-up rate of 92.6%.

### Survival analysis

**Fig. 1:**
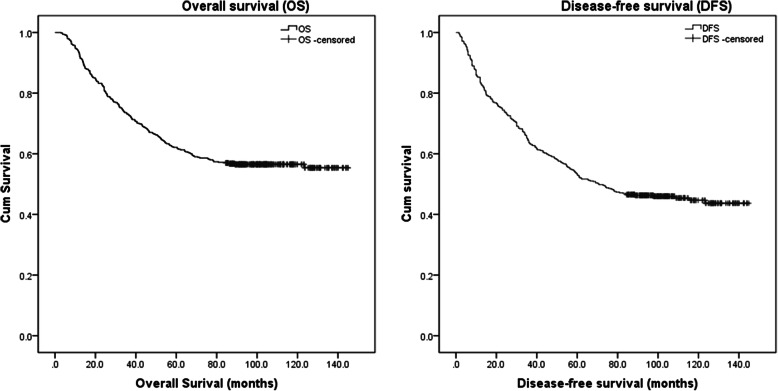
Survival curves for pT_1–3_N_0_M_0_ esophageal squamous cell carcinoma patients after surgery alone

At the follow-ups for 1, 2, 3, 4, 5 and 8 years, the rates of OS obtained were 93.2, 82.2, 73.0, 66.8, 62.1 and 56.5%, and the rates of DFS were 83.4, 73.8, 63.7, 59.0, 53.1 and 46.3%, respectively (Fig. 1). Moreover, at the follow-ups for 1, 3, 5 and 8 years, the rates of RFS were 85.7, 68.0, 58.3 and 51.8%, and the rates of LRFS were 87.5, 73.3, 65.6 and 59.9%, respectively (Table 2).

**Table 2:** Factors associated with OS or DFS according to univariate analysis for pT_1 − 3_N_0_M_0_ ESCC patients after surgery alone

Factors	Number	OS		p	DFS		p
		3-year	5-year		3-year	5-year	
Gender							
Male	303	71.3	59.7	0.162	62.4	53.1	0.366
Female	185	75.7	65.9		65.9	53.0	
Age							
≤ 65 years	339	74.0	65.2	0.019	66.1	56.0	0.064
> 65 years	149	70.5	55.0		54.8	46.3	
Site of lesion							
Upper	61	57.4	47.5	0.033	44.3	36.1	0.008
Middle	344	74.1	62.5		64.2	53.2	
Lower	83	79.5	71.1		75.9	65.1	
Length of lesion							
< 5 cm	259	72.6	61.8	0.907	63.3	51.7	0.558
≥ 5 cm	229	73.4	62.4		64.2	54.6	
SLNs in CT							
No	373	76.4	66.0	0.002	67.6	56.8	0.001
Yes	115	61.7	49.6		51.3	40.9	
Dissected LNs							
< 12	337	68.8	58.5	0.034	58.8	51.0	0.149
≥ 12	151	82.1	70.2		74.8	57.6	
Differentiation							
Well/moderate	450	73.3	62.2	0.163	64.2	53.3	0.323
Poor	38	68.3	55.3		57.9	50.0	
pT							
pT_1_	102	89.2	84.3	< 0.00	82.4	71.6	< 0.001
pT_2_	126	77.0	65.1		65.1	50.8	
pT_3_	260	64.6	51.9		55.8	46.9	

*Abbreviations and definitions*: *OS* overall survival, *DFS* disease-free survival, *ESCC* esophageal squamous cell carcinoma, *dissected LNs* the number of dissected lymph nodes at the time of surgery, *SLNs in CT* small lymph nodes in mediastinum (diameter < 1 cm) in CT image prior to surgery

Univariate analysis showed that site of the lesion, SLNs in CT, and pT stage were associated with OS, DFS, RFS and LRFS (*p* < 0.05); however, associations with patient age and dissected LNs were only established for OS, not for other survival parameters. Gender, size of the tumor, and histopathological differentiation of ESCC were not associated with OS, DFS, RFS or LRFS (*p* > 0.05; Table 2). Multivariate Cox analysis revealed that, patient age, the site of the lesion, SLNs in CT, dissected LNs and pT stage were independent factors for OS (Tables 3); the site of the lesion, SLNs in CT, and pT stage were independent factors for DFS, RFS and LRFS (Tables 4).

**Table 3:** Factors associated with OS according to multivariate Cox analysis

Factors	Groups	HR (95% CI)	p
Age	≤ 65	1.000	
	> 65	1.339 (1.011–1.772)	0.042
Site of lesion	Upper	2.237 (1.353–3.699)	0.002
	Middle	1.380 (0.929–2.052)	0.111
	Lower	1.000	
SLNs in CT	No	1.000	
	Yes	1.554 (1.152–2.095)	0.004
Dissected LN	< 12	1.502 (1.105–2.041)	0.009
	≥ 12	1.000	
pT	pT_1_	1.000	
	pT_2_	2.712 (1.624–4.529)	< 0.001
	pT_3_	3.710 (2.309–5.962)	< 0.001

*Abbreviations and definitions*: *OS* overall survival, *HR* hazard ratio, *CI* confidence interval, *dissected LNs* the number of dissected lymph nodes at the time of surgery, *SLNs in CT* small lymph nodes in mediastinum (diameter < 1 cm) in CT image prior to surgery

**Table 4:** Factors associated with DFS, RFS and LRFS according to multivariate Cox analysis

Factors	Groups	DFS		RFS		LRFS	
		HR (95% CI)	p	HR (95% CI)	p	HR (95% CI)	p
Site of lesion	Upper	2.214 (1.409–3.479)	0.001	2.560 (1.552–4.222)	< 0.001	2.251 (1.295–3.911)	0.004
	Middle	1.384 (0.972–1.970)	0.071	1.551 (1.040–2.314)	0.031	1.434 (0.930–2.211)	0.103
	Lower	1.000		1.000		1.000	
SLNs in CT	No	1.000		1.000		1.000	
	Yes	1.555 (1.187–2.036)	0.001	1.580 (1.185–2.106)	0.002	1.577 (1.145–2.174)	0.005
pT	pT_1_	1.000		1.000		1.000	
	pT_2_	1.944 (1.306–2.891)	0.001	1.861 (1.222–2.834)	0.004	1.954 (1.204–3.171)	0.007
	pT_3_	2.268 (1.583–3.249)	< 0.001	2.102 (1.433–3.082)	< 0.001	2.316 (1.491–3.600)	< 0.001

*Abbreviations and definitions*: *DFS* disease-free survival, *RFS* recurrence-free survival, *LRFS* locoregional recurrence-free survival, *HR* hazard ratio, *CI* confidence interval, *SLNs in CT* small lymph nodes in mediastinum (diameter < 1 cm) in CT image prior to surgery

### Recursive partitioning analysis scores

A recursive partitioning analysis (RPA) model was used to predict the survival of ESCC patients in previous studies [15–17]. Based on the five independent prognostic factors for OS in our study, risk levels were further stratified as 0, 1 and 2 in accordance with the Cox analysis. The total RPA score was calculated for each patient using the following factors: gender (female = 0, male = 1), site of the lesion (lower or middle segment = 0, upper segment = 1), SLNs in CT (no = 0, yes = 1), dissected LNs (≤ 12 = 0, > 12 = 1) and pT stage (pT_1_ = 0, pT_2_ = 1, pT_3_ = 2).

**Fig. 2:**
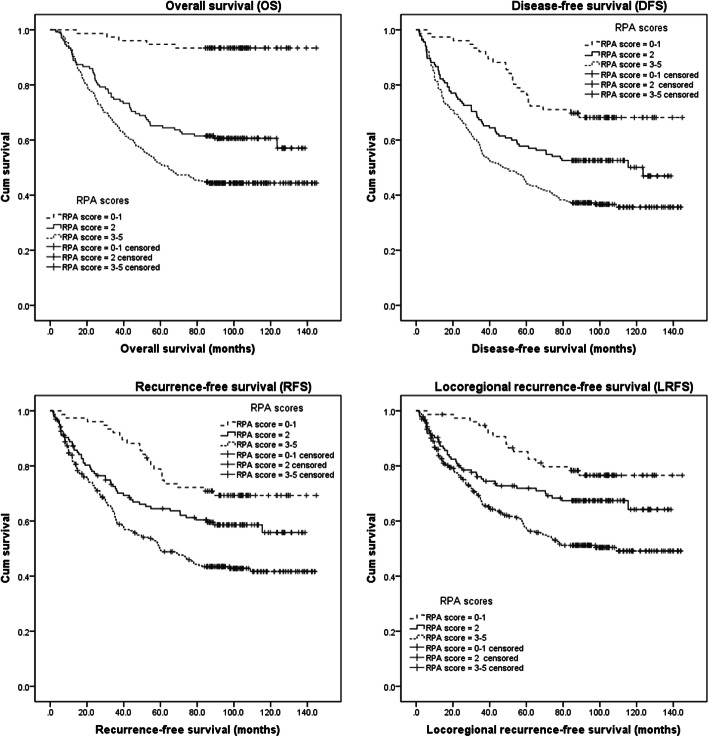
Survival curves based on recursive partitioning analysis (RPA) scores for pT_1–3_N_0_M_0_ esophageal squamous cell carcinoma patients after surgery alone

Once with RPA scores were determined, the patients were first assigned into six groups with scores of 0 (18 patients), 1 (58 patients), 2 (135 patients), 3 (154 patients), 4 (97 patients) and 5 (26 patients). Patients were then further classified according to risk level: low-risk group (RPA score: 0–1, 76 cases), medium-risk group (RPA score: 2, 135 cases), and high-risk group (RPA score: 3–5, 277 cases). The rates of OS, DFS, RFS and LRFS were significantly different among all three groups (*p* < 0.001; Fig. 2 and Table 5).

**Table 5:** Survival rates according to various RPA scores

	Class (RPA scores)	1	Year 3	5	8	*x*^2^	p
**OS**	Class 1 (0–1)	100	97.4	94.7	93.4	50.845	< 0.001
	Class 2 (2)	91.1	74.8	65.2	60.6		
	Class 3 (3–5)	92.4	65.3	51.6	44.4		
**DFS**	Class 1	97.4	92.1	77.6	68.2	28.295	< 0.001
	Class 2	85.2	65.9	57.8	52.6		
	Class 3	78.7	54.9	44.0	37.2		
**RFS**	Class 1	97.4	92.1	78.9	69.3	21.821	< 0.001
	Class 2	87.3	70.9	64.5	58.6		
	Class 3	81.7	59.6	49.3	43.4		
**LRFS**	Class 1	98.7	94.7	85.2	76.6	20.705	< 0.001
	Class 2	88.7	75.3	71.9	67.4		
	Class 3	83.7	66.1	56.7	51.2		

*Abbreviations*: *RPA* recursive partitioning analysis, *OS* overall survival, *DFS* disease-free survival, *RFS* recurrence-free survival, *LRFS* locoregional recurrence-free survival

## Discussion

The optimal surgical technique for the curative treatment of patients with pN_0_ esophageal cancer has remained controversial. Currently, the procedures of choice include R0 esophagectomy with two-field lymphadenectomy based on evidence of early submucosal infiltration and early-stage lymphatic dissemination of the cancer, both of which pose challenges for the long-term outcomes of the surgery.

Two studies have already shown that the five-year OS rates were 51.2% for pT_1–3_N_0_M_0_ [3] and 52.9% for stage pN_0_ [4] ESCC patients who underwent two-field surgery without neoadjuvant or adjuvant chemoradiation therapies. Moreover, another study based on 2588 patients with pT_1–3_N_0_M_0_ EC [18] further reported that the postoperative five- and 10-year OS rates were 67.1 and 57.6%, respectively, and the cause-specific survival (CSS) rates were 80.8 and 77.7%, respectively. In this retrospective study, 488 pT_1–3_N_0_M_0_ ESCC patients underwent R0 esophagectomy with two-field lymphadenectomy in the absence of either preoperative neoadjuvant or postoperative adjuvant therapy. At 3, 5 and 8 years following the surgery, the OS rates were 73.0, 62.1 and 56.5%, and the DFS rates were 63.7, 53.1 and 46.3%, respectively.

Taken together, even with the slight variation in five-year OS rates among the different studies, the OS remains at approximately 50% for radical esophagectomy coupled with two-field lymphadenectomy without significant improvement for different patient populations or different surgical teams. Chen et al. [19] reported that three-field lymphadenectomy results in five and 10-year OS rates of 71.3 and 57%, respectively, for pT_1-4a_N_0_M_0_ ESCC patients, which were significantly improved compared to the two-field procedure, indicating early lymphatic dissemination might not be confined to regional LN surrounding the lesion. However, Shao et al. [20] showed that the OS was not different between two-field and three-field LN dissection for pN_0_ ESCC patients. Taken together, the use of three-field LN dissection to potentially improve OS should be further investigated.

To better understand the risk factors associated with the OS of ESCC patients after two-field lymphadenectomy, survival analyses with univariate as well as multivariate Cox regression models were used in the current study. The results indicated that pT_1–3_N_0_M_0_ ESCC patients who presented with neoplasms in the upper-thoracic segment had the worst OS and DFS in comparison to patients with tumors in the middle or lower thoracic segments. Although this was similar to previous studies of EC patients who were mainly operated on through the left thoracic approach [5, 21], it was also contrary to several studies that have demonstrated that the site of the lesion is not associated with prognosis for ESCC patients who are operated on using the right thoracic approach [3, 4, 19, 22]. Therefore, additional studies are required to investigate and validate the true influence that the site of the lesion has on the OS of patients.

Prospectively, preoperative CT assessments may allow for the visualization of potential LN metastases, which can help to determine the surgical approach and procedure that will result in the best long-term prognosis for the patient. Because the surgical method used in this study was the Sweet procedure, this might have led to insufficient mediastinal LN dissection and subsequent potentiation of metastasis of SLNs. As such, the present study identified SLNs in CT prior to surgery as an independent risk factor associated with OS and DFS.

Several studies have suggested that OS was not significantly impacted in patients with middle or lower thoracic EC who underwent a Sweet or Ivor Lewis esophagectomy [23–27], while the study from Ma Q et al. [26] showed that the three- and five-year rates of CSS and OS were better for the pN_0_ ESCC patients with left transthoracic approaches (eg. Sweet) compared to those with right transthoracic approaches (eg. Ivor Lewis), indicating that the location of cancer impacts the long-term survival of patients.

The pT stage was another important independent factor for OS and DFS in our study as well as other studies. The studies by either Xie et al. [22] and Gao et al. [18] reported that the five-year OS rates were 75.1 and 77.8% for pT_1_, 50.4 and 54.2% for pT_2,_ and 37.0 and 34% for pT_3_ ESCC patients with pN_0_ after two-field surgery, respectively. Chen et al. [19] reported that the five- and 10-year OS rates were 83.8 and 71.9% for pT_1_N_0_M_0_, 78.8 and 67.4% for pT_2_N_0_M_0_, 67.8 and 51.1% for pT_3_N_0_M_0_ ESCC patients after three-field surgery, respectively. Our results also showed that the pT stage was associated with RFS and LRFS, suggesting that early diagnosis and surgical interventions are important for the long-term prognosis of EC patients.

Two-field lymphadenectomy was chosen as the routine procedure for the surgical management of ESCC, and numerous studies have already shown that the number of dissected LNs can influence the long-term mortality of ECSS patients. Xie et al. [22] reported that the five-year OS rates for dissected LN numbers of 0–14, 15–19, 20–24, and ≥ 25 in pT_1–3_N_0_M_0_ ESCC patients were 28.5, 47.7, 56.4 and 60.4% after surgery, respectively. Yang et al. had demonstrated the five-year OS rates for pN_0_ ESCC patients with dissected LN numbers of < 6, 6–9, 10–17, and ≥ 18 were 40.8, 50.6, 55.9 and 71.4%, respectively [28]. However, Altorki et al. reported that only over 40 LNs dissected could produce significantly better OS rates, compared with less than 16 LNs dissected [13]. A single study [29] has aggressively claimed that the number of dissected LNs is not associated with the OS of pN_0_ EC patients. Our study found that the five-year OS rates for patients with dissected LNs < 12 and ≥ 12 were 58.5 and 70.2%, respectively. While these data are similar to some of the previous studies, it also suggests that the optimal number of dissected LNs to improve long-term outcomes should be further investigated.

In our study, patient age was associated with OS but not with DFS for pT_1–3_N_0_M_0_ ESCC patients. Chen et al. [19] also showed that the five-year OS rate for pN_0_ ESCC patients was 76.5% for those younger than 60 years of age and 63.3% for those 60 years of age or older. Other studies [4, 25, 29] have also shown age as an independent factor for OS in ESCC patients.

Recently, nomogram and RPA scores have been used to predict the survival of ESCC patients following surgery and to stratify postoperative patients into varying risk groups. Many studies from various clinic groups have reported their methods to establish nomograms to classify the risk level of the ESCC patient. Zheng et al. [15] selected five independent predictors of OS (gender, age, dissected LNs, pT, and pN status) to evaluate clinical nomograms in ESCC patients after surgery. Yu et al. [16] used the LN metastatic ratio and adjuvant therapy to construct their nomogram and RPA to classify patients with IIB-III ESCC. Ni et al. [17] attempted to include patient age, pTMN stage, and management modalities to classify ESCC. Duan et al. [30] used five independent prognostic variables to build the nomograms to predict DFS and OS of ESCC patients undergoing postoperative chemo and radiation therapy. This prognostic nomogram provided an individualized risk estimate of survival in patients after esophagectomy followed by postoperative chemoradiation therapy. Deng et al. [24] used eight independent risk factors to build the nomogram to predict the OS of patients with pT_1_N_+_/T_2-4a_N_0–3_, M_0_ ESCC after surgery. The prognostic efficacy of the nomogram in the training and validation cohorts was significantly greater than that of the American Joint Committee on Cancer (AJCC) staging system.

We classified pT_1–3_N_0_M_0_ ESCC patients into three classes (low, middle, and high risk) according to the RPA scores; the OS, DFS, RFS and LRFS were significantly different among the three classes. For the low-risk group, the five-year OS was > 90% and the recurrence rate was very low. Therefore, postoperative adjuvant therapy is not needed. For the middle-risk group, the five-year OS was approximately 65% and postoperative adjuvant therapy should be considered. For the high-risk group, the five-year OS was approximately 50% and postoperative adjuvant therapy should be strongly recommended. Several studies have shown the value of postoperative adjuvant therapy in pN_0_ EC patients [18, 19, 31, 32]; however, the adverse effects related to postoperative adjuvant therapy are a considerable hurdle for patients to undertake therapy. Therefore, it may be more reasonable to select postoperative adjuvant therapy based on the likelihood of postoperative survival and/or recurrence in ESCC patients.

Several pitfalls can be found in our study. Firstly, this retrospective case-matched study was conducted with patients from a single-center. Therefore, the possibility of selection bias could not be entirely excluded despite the use of the multivariate analysis. Secondly, most of the patients in our study underwent the left thoracic approach for R0 esophagectomy and two-field lymphadenectomy with a median number of 10 dissected LNs. As such, the data and subsequent conclusions might only be suitable for similar patients. Thirdly, the details of recurrence and salvage therapy were not shown in this study. Salvage therapy might impact the OS of our patients.

## Conclusions

In this study, the site of the lesion, SLNs in CT before surgery, and pT stage were established as the independent risk factors that negatively impact OS, DFS, RFS and LRFS, while the age of the patient and the number of dissected LNs were additional risk factors for the OS in pT_1–3_N_0_M_0_ ESCC patients after two-field surgery alone. With these risk factors, a practice-oriented method was proposed with RPA scores, which stratifies the postoperative patients into three degrees of risk: low, medium, and high. This stratification provides guidance regarding the importance of postoperative adjuvant therapy to improve OS.

## Data Availability

The dataset(s) supporting the conclusions of this article is (are) included within the article.

## Abbreviations

CSS: Cause-specific survival
CT: Computed tomography
DFS: Disease-free survival
DM: Distant metastases
EC: Esophageal cancer
ESCC: Esophageal squamous cell carcinoma
LN(s): Lymph nodes
LRFS: Locoregional recurrence-free survival
LRR: Locoregional recurrence
OS: Overall survival
RFS: Recurrence-free survival
RPA: Recursive partitioning analysis
SLNs: Small lymph node
